# The transition into veterinary practice: Opinions of recent graduates and final year students

**DOI:** 10.1186/1472-6920-11-64

**Published:** 2011-09-22

**Authors:** Susan M Rhind, Sarah Baillie, Tierney Kinnison, Darren J Shaw, Catriona E Bell, Richard J Mellanby, Jenny Hammond, Neil PH Hudson, Rachel E Whittington, Ruth Donnelly

**Affiliations:** 1 Royal (Dick) School of Veterinary Studies, University of Edinburgh, Easter Bush Veterinary Centre, Roslin, Midlothian, UK; 2 Royal Veterinary College, Hawkshead Lane, North Mymms, Hatfield, Hertfordshire, UK; 3 University of Glasgow, Faculty of Veterinary Medicine, 464 Bearsden Road, Glasgow, UK; 4 University of Edinburgh Careers Service, 33 Buccleuch Place, Edinburgh, UK

## Abstract

**Background:**

The transition from veterinary student to member of the veterinary profession is known to be challenging. This study aimed to determine and compare the opinions of final year veterinary students and recent graduates on graduate attributes that ease this transition.

**Methods:**

The study was carried out across 3 veterinary schools in the United Kingdom. Paper based or electronic surveys were used. Final year students in the 3 schools were surveyed either electronically (school A) or on paper (schools B and C). Student cohort sizes were 112, 227 and 102 respectively. Recent graduates were contacted either at a reunion event (school A) or electronically from database records (school B and school C). Cohort sizes of contacted graduates were 80, 175 and 91 respectively. Respondents were asked to rate 42 individual attributes on a 5 point Likert scale. Focus groups with final year students and recent graduates and telephone interviews with recent graduates were carried out. Data were analysed by two researchers through a combination of manual coding and thematic analysis. Data were grouped into broad themes then sorted into narrower themes. Data were then searched for counter examples.

**Results:**

Response rates for final year students were 34% (school A), 36% (school B) and 40% (school C). Response rates for recent graduates were 56% (school A), 20% (school B) and 11% (school C). There was a high level of agreement between the cohorts with respect to communication skills, problem solving and decision making skills, recognition of own limitations and the ability to cope with pressure all rated unanimously important or very important. Business acumen, knowledge of veterinary practice management and research skills were the 3 attributes ranked at the bottom of the list. Nine attributes were identified with a significantly different (p < 0.05) ranking between the cohorts. Final year students ranked veterinary clinical knowledge, knowledge of veterinary public health and zoonotic issues, veterinary legislation and veterinary practice management, commitment to continuing professional development and ability to evaluate information higher than recent graduates. Recent graduates ranked the attributes of integrity, friendliness and compassion higher than final year students.

**Conclusions:**

Recent graduates and final year students rate highly the attributes which help foster the client/veterinarian relationship. Recent graduates reflect that a focus on knowledge based attributes is less important once in practice when compared to final year. The study confirms the importance to recent graduates and final year students of attributes considered as non-technical in the transition to working in the veterinary profession.

## Background

A central requirement of the veterinary curriculum is to provide new graduates with the necessary skills to be a successful clinician. In addition to experiences within the curriculum, all veterinary students in the United Kingdom are required by the Royal College of Veterinary Surgeons (RCVS) [1] to spend a total of 38 weeks spread across preclinical and clinical placements (extra-mural studies/EMS) during the veterinary degree programme [2] and cannot achieve membership of the RCVS without doing so. To guide curricula, a list of 'day one competences' has existed for a number of years defined by the RCVS [1]. These competences are divided into 3 categories: General Professional Skills and Attributes, Underpinning Knowledge and Understanding and Practical Competences In addition to these day one competences, the RCVS implemented the 'postgraduate development phase', or PDP, in 2007 [3]. The PDP aims to guide new graduates as they develop within the profession to a set of 'Year One Competences' which are presented in the same overarching categories described above for the day one competences. This is a particularly important development for veterinary graduates since unlike medical graduates, there is no standardised career structure post-graduation.

In addition to the development of these professional competency statements, lists or frameworks of 'graduate attributes' have become increasingly present in the higher education community over recent years. It has been emphasised elsewhere [4] that these attributes represent the skills and abilities 'needed to thrive in a job'. This trend has emerged in parallel with discussions on employability. Employability has been defined as 'a set of achievements - skills, understandings and personal attributes - that make graduates more likely to gain employment and be successful in their chosen occupations' [5]. A link between distress or dissatisfaction in the veterinary workplace and inadequate development of graduate attributes has been suggested [6]. This is especially topical given the recognition that there is growing evidence of poor mental health amongst some members of the profession [7], a relatively high incidence of suicide [8], high attrition rates from early years in practice [9] and considerable workplace stress [10].

When considering discipline specific graduate attributes, important information can be obtained from recent graduates as this group have most recently had to deal with the transition from student to clinician. Gilling and Parkinson [11] as part of a study examining the outcomes of a veterinary degree programme identified the importance and indeed pivotal nature of the first year in practice. A study by Walsh et al [12] explored final year student and graduate opinions (from one North American school) on a series of graduate attributes. Ratings across the two groups were similar with subtle differences for example in opinion on the importance of competence in information technology which was considered more important by final year students [12].

The aim of this project was to establish the attributes which recent veterinary graduates (1-2 years after graduation) believe serve to ease the transition from the undergraduate student environment to working as a member of the profession. An additional aim was to compare these opinions to those of final year veterinary students. As with all UK veterinary schools, the students sit a final year examination examining competences across species and disciplines consistent with the RCVS day one competences described earlier [1].

## Methods

The study was carried out across 3 veterinary schools in the United Kingdom. All 3 schools have similar curricula and a final year focussed on practical work in clinics and support services.

The three veterinary schools involved in the project are designated school A, school B and school C.

The survey developed for use in this project was a version of the University of Edinburgh graduate attributes framework [13] with modifications specific to the veterinary profession guided by the RCVS day one competences [1]. The attributes were clustered into 6 categories: subject specific, general skills and attributes, research and enquiry, personal and intellectual autonomy, communication skills and personal effectiveness. Respondents were asked to rate individual items on a 5 point Likert scale (not at all important, not important, indifferent, important, very important). Recent graduates in addition were asked about their current type of work. Surveys were developed by an iterative process between the authors and piloted with final year veterinary students in the cohort preceding this study in school A. The final year student and recent graduate surveys are available as additional files 1 and 2 respectively.

Distribution of questionnaires across the schools differed according to local decision on which methods were most likely to give the best response rates given timetables (undergraduates) and alumni events (recent graduates). The methods used in each school are summarised in Table 1 with additional detail given below.

**Table 1:** Summary of response rates and data collection methods for each cohort and each school

School	Cohort (size)	Recruitment mode	Survey mode	Responses (%)
A	FYS (112)	Group presentation + electronic reminders	Electronic	38/112 (34)
	
	RG (80)	Graduate reunion	Paper	45/80 (56)

B	FYS (227)	During tutorial	Paper	82/227 (36)
	
	RG (175)	Electronic from school records	Electronic	35/175 (20)

C	FYS (102)	During practical class	Paper	41/102 (40)

	RG (91)	Electronic from school records	Electronic	10/91 (11)

FYS: Final Year Student RG: Recent Graduate

### School A

Student survey. This was issued electronically and was live for 4 weeks with an electronic reminder sent half way through this period.

Recent graduate survey. Recent graduates attended a 'graduate reunion' where a paper version of the survey was distributed.

### School B

Student survey. Final year students were asked to complete a paper copy of the survey during a tutorial session.

Recent graduate survey. Recent graduates were contacted electronically via a global e-mail list held by the school.

### School C

Student survey: A paper version of the survey was made available during practical sessions. A short spoken introduction was given to ensure each group was aware of the nature of the survey, and a request was made for students to complete the survey if they wished. Surveys were completed during or immediately after the session.

Recent graduate survey: A list of postal contact details was provided by the alumni office. Up to date contact details for 80 of the 99 students in the cohort were available. Paper surveys along with a standard consent form were sent to all these addresses. A deadline for return of surveys was given as 6 weeks from receipt.

Focus groups and interview participants were randomly selected from those indicating on the questionnaire a willingness to participate further in the study.

The project was approved by the College of Medicine and Veterinary Medicine Research Ethics Committee (University of Edinburgh).

### Statistical analysis

A two-step approach to the analysis was adopted. Firstly, for each attribute the association between the response 'very important' and 'important' was compared to the remaining three responses ('not important at all', 'not important', 'indifferent') in the final year students compared to the recent graduates and was assessed by standard Fisher Exact tests. Secondly, Fisher Exact tests were also used for each attribute to compare the proportion of responses of 'very important' to the other categories ('not important at all', 'not important', 'indifferent' and 'important') in the final year students compared to the recent graduates. These two analyses were carried out in recognition of the fact that participants were not trained in the distinction between the different Likert scale categories. In all cases a p < 0.05 was taken to indicate statistical significance. In addition Odds ratios (and associated exact binomial 95% confidence intervals) were also calculated.

Analysis was carried out by investigating the percentage of each group who ranked the attributes as very important. Additional analysis grouped the ranking of important and very important together and again compared the responses across both groups. Analysis of results across the two groups was carried out in this way in recognition of the ordinal but non interval nature of the data [14].

### Qualitative data analysis

Interviews and focus group discussions were transcribed and analysis carried out through a combination of manual coding and thematic analysis in Nvivo. Transcripts were read through several times in order to become familiar with the data and to pick out common themes [15]. The data were initially grouped into broad themes in order to provide a solid but not too specific starting point [16]. Further to this, the data were sorted into a larger number of narrower themes, some of which were later enveloped as sub-themes in larger overall themes. With the major themes established, the data were searched again for counter examples to the major trends in order to see how widespread a view they represented and for other themes that may have previously been missed. Validation of the themes was carried out by a second researcher. Our approach was based on Charmaz's (2003) constructivist perspective on qualitative data [17].

## Results

A total of 161 final year veterinary students completed the surveys across the 3 schools and 90 recent graduates (1-2 years post-graduation) from the same 3 schools. The percentage response rate of those surveyed is shown in Table 1.

The majority of the graduates were in either mixed or small animal practice with 4 in exclusively equine practice and 3 in exclusively farm animal practice.

Nine final year students took part in 2 separate focus groups while 8 recent graduates participated in telephone interviews.

Given the variable response rates across the schools, a decision was taken to analyse the data by group only and not break this down to compare across schools. The possibility of different curricula affecting responses was not explored in this project given that all curricula are addressing the day one competencies [1] and the questionnaire did not specifically ask questions relating to the particular curriculum a student had experienced. Rather the focus was on exploring each group's opinions on the attributes which they considered important in the transition into the profession from their standpoint - either of student or graduate.

### Relative ranking of attributes

Table 2 shows the list of all attributes in the questionnaire ordered by the percentage of respondents who ranked them as 'very important'. There was a high level of agreement between the cohorts with communication skills, problem solving and decision making skills, recognition of own limitations and the ability to cope with pressure all rated unanimously important or very important. Business acumen, knowledge of veterinary practice management and research skills were the 3 attributes ranked at the bottom of the list.

**Table 2:** List of all attributes in the questionnaire ordered by the percentage of respondents who ranked them as 'very important'

Attribute	RG	Attribute	FYS
Communication with clients and the public	88	Communication with clients and the public	88

Communication with colleagues	81	Communication with colleagues	83

Recognising own limitations & know when to seek advice	80	Practical skills	82

Listening skills	80	Recognising own limitations & knowing when to seek advice	81

Practical skills	78	Listening skills	77

Ability to cope with pressure	71	Problem solving	75

Problem solving	70	Veterinary clinical knowledge	74

Interpersonal and teamwork skills	67	Decision making	70

Decision making	66	Ability to cope with pressure	67

Compassion	62	Ability to handle difficult situations	65

Confidence	61	Interpersonal and teamwork skills	63

Integrity	57	Confidence	62

Patience	57	Politeness	47

Ability to handle difficult situations	54	Compassion	46

Veterinary clinical knowledge	53	Patience	46

Friendliness	51	Ability to cope with uncertainty	46

Flexibility in adapting to new situations	49	Integrity	44

Ability to cope with uncertainty	49	Flexibility in adapting to new situations	44

Negotiation skills	47	Time management skills	42

Decisiveness	44	Decisiveness	41

Politeness	43	Ethical awareness	41

Thinking creatively and independently	40	Negotiation skills	40

Time management skills	33	Thinking creatively and independently	39

Ethical awareness	32	Friendliness	37

Report writing and record keeping skills	30	Organisational skills	36

Organisational skills	28	Attention to detail	34

Attention to detail	23	Report writing and record keeping skills	33

Capacity for self-audit	23	Knowledge of veterinary legislation	27

Presentation skills	20	Knowledge of vet public health/zoonotic issues	27

Numeracy skills	19	Capacity for self-audit	25

Commitment to CPD	16	Numeracy skills	23

Analytical skills	16	Presentation skills	20

IT/computer literacy	16	Commitment to CPD	20

Leadership skills	14	Analytical skills	19

Knowledge of vet public health/zoonotic issues	13	Professional appearance	17

Ability to evaluate information	11	Ability to evaluate information	16

Professional appearance	11	Leadership skills	15

Knowledge of veterinary legislation	8.9	Knowledge of underpinning science	12

Knowledge of underpinning science	5.6	IT/computer literacy	11

Business acumen	4.4	Business acumen	5.7

Knowledge of veterinary practice management	2.2	Knowledge of veterinary practice management	5.6

Research skills	2.2	Research skills	1.2

(RG- recent graduates, FYS - final year students). Values are given to two significant figures.

### Comparison of cohort responses

For 33 of the attributes the difference in their ranking as very important or very important/important was not significant (P > 0.083). The remainder of the data are summarised in Table 3 Table 4 and Table 5 which present the data for the 9 attributes where a p value of < 0.05 was identified.

**Table 3:** Attributes considered significantly more important to final year students than recent graduates

Attribute	Final Year Student	Recent Graduate	P value	Odds ratio (95% CI)
Commitment to CPD	82	71	**0.047**	0.54 (0.29,0.99)

Ability to evaluate information	84	61	**0.001**	0.3 (0.17,0.55)

Knowledge of veterinary practice management	65	48	**0.013**	0.51 (0.3,0.87)

Percentage response rates for final year students and recent graduates for the listed attributes with categories very important and important grouped together. Values are given to two significant figures.

**Table 4:** Attributes considered significantly more important to final year students than recent graduates

Attribute	Final Year Student	Recent Graduate	P value	Odds ratio (95% CI)
Veterinary clinical knowledge	74	53	**0.001**	0.4 (0.23,0.69)

Knowledge of veterinary public health/zoonotic issues	27	13	**0.015**	0.42 (0.21,0.84)

Knowledge of veterinary legislation	27	8.9	**0.001**	0.26 (0.12,0.58)

Percentage response rates in the category 'very important' for final year students and recent graduates for the listed attributes. Values are given to two significant figures.

**Table 5:** Attributes considered significantly more important to recent graduates than final year students

Attribute	Final Year	Recent Graduate	P value	Odds ratio (95% CI)
Integrity	44	57	**0.046**	1.7 (1.01,2.86)

Friendliness	37	51	**0.027**	1.81 (1.07,3.05)

Compassion	46	62	**0.014**	1.94 (1.14,3.28)

Percentage response rates in the category 'very important' for final year students and recent graduates for the listed attributes. Values are given to two significant figures.

Analysing the data in this way showed that a higher percentage of final year students ranked the attributes of commitment to continuing professional development (CPD), ability to evaluate information and knowledge of veterinary practice management as very important or important than recent graduates (Table 3). In addition a higher percentage ranked veterinary clinical knowledge, knowledge of veterinary public health/zoonotic issues and knowledge of veterinary legislation as very important than the recent graduates (Table 4). A higher percentage of the recent graduates ranked integrity, friendliness and compassion as very important (Table 5).

### Qualitative data analysis

In order to explore the quantitative results further, 9 final year students took part in 2 separate focus groups while 8 recent graduates participated in telephone interviews. Schedules were developed to explore participants' views on the attributes ranked at the top (5) and bottom (3) of the list (42). Further to this, 3 recent graduates were also asked for their opinions on the 9 attributes where there was significant difference between the response of final years and recent graduates. Common themes are illustrated by relevant quotes with counter examples if available. The source of the data is indicated by respondent (recent graduate - RG; final year student - FYS and numbered to identify different participants i.e. FYS 1-9; RG 1-8).

### Exploration of the top ranked attributes

The following themes relating to the top ranked attributes emerged.

### Communication Skills

Theme: the client relationship

"It's a key part to the job because the animal comes with an owner attached to it. You have to speak to the owner and you can't get away without it, it plays a part in getting a history and finding out what's going on with the animal." RG5

"...the only way you can glean information on your patient or....in fact you have to be able to speak with your clients because otherwise you are not going to get the necessary information that you need to actually treat the patient." FYS9

"If you can't build up that rapport with the clients to start with, they're not going to come back, they're not going to trust you". RG4

"Because if you don't get on with people, it doesn't matter how good you are with their animals, they're not going to like you and they're not going to come back and see you." RG6

Theme: communication and the curriculum

"[the course] was good but it still felt like a setup situation. So final year I think has been good with clients because you do get a lot of sort of free rein with the consults really" FYS8

"I found that the communication skills workshops were really useful, but I've heard varying opinions on them, other people didn't find them so useful" FYS1

### Recognising limitations and knowing when to seek advice

Theme: the link with confidence

"you have to realise that there are things you cannot do as a new graduate and you do not want to put any animal's life in danger or anything like that or miss something significant because you are not certain. I think you do have to recognise that there are things you just cannot do at this stage or you are uncertain how to do at this stage and be willing, be willing to ask for help for it when you are at that point" FYS9

"You don't want to feel bad; look bad... don't want people to laugh at you... if you say something stupid it just reflects so badly, that I think it takes some of the confidence out of people." RG2

### Practical Skills

Theme: the need for more experience

"Certainly every graduate would like to have better practical skills, because they are what you get judged on and they are where your own confidence comes from." RG4

"I guess a lot of practitioners don't recognise that the years are a lot larger now and when they came through they probably did more practical stuff, than now, people don't get to do as much" FYS2

Although not all participants agreed with this as illustrated below.

"most people I have spoken to say you will do more practical things in your first couple of weeks of practice than you have ever done in EMS" FYS8

Theme: the variability of extramural studies (EMS)

"....it utterly depends on how much they let you do on EMS, which is really hit and miss." RG6

"there are a lot of places that you choose either by convenience or by basically wherever you can get in and then you go and you realise it is not actually a beneficial experience at all." FYS9

"some people that had done absolutely no surgery and some people that had done a fair amount, because they'd gone back routinely to the same placement and got a relationship with them and been allowed to do more than they would have otherwise." RG8

### Exploration of the bottom ranked attributes

The following themes relating to the bottom ranked attributes emerged.

### Research Skills

The focus groups were used as an opportunity to explore students and graduates understanding of the term with a clear theme emerging that the reason for the relatively low ranking (Table 1) linked to relating the term to laboratory research work as indicated by the quotes below.

Theme: lack of relevance

"the working in the lab side of things, that clearly is not very relevant to most graduates." RG5

"I know people who are interested in research and they are either going to go into practice and still do some research or go straight into doing research but the majority of people I know do not want to do research." FYS9

Some participants did however recognise the wider meaning of the term

"I would definitely rate it higher, it's really important, but I would consider it under the title 'Problem solving abilities" RG7

### Business Acumen

Although several participants could understand the relatively low ranking of this attribute, a series of quotes also indicated an understanding of why it is important and indeed that it should be rated higher.

Theme: explanation of why the attribute was rated comparatively low

"I don't think a lot of graduates would like to think of themselves as earning money for something and they don't like to think of themselves as part of a business." RG3

"And when I decided I wanted to be a vet, I did not think I want to go into business, I thought I want to talk to clients and work in a team and that is what is important in a job to me" FYS8

Theme: understanding of why the attribute is important

"You have to be aware of what things cost though, so that you can justify it to the clients .... and if you can explain to them why, then it makes sense" FYS3

Theme: conflict between ethical and welfare obligation and economics

"A lot of people think that you are the caring vet, who's only concerned about the welfare of their animal, and that it's really mean of you to charge them!" FYS3

### Practice Management and relevance to practice

Theme: more important with time and experience

"As a new grad, you are so overwhelmed with all of the immediate and critical demands on your time and your knowledge... you're so grateful that it's not something you have to worry about." RG7

"so maybe the reason people have said it is not important is because it is something you do pick up when you are working there, how it works" FYS7

### The changing views of recent graduates and final year students

Theme: Knowledge is less important than knowing where to find information

"you find that in practice you can always read a book or phone up the referral place for some advice or just work out what further tests to do. So certainly I was a lot more worried about clinical knowledge at uni than I necessarily was when I came out." RG6

"I wouldn't think twice about getting a book out and double-checking things. I don't feel I have to know everything off the top of my head." RG8

### Limitations

We appreciate the limitations of the Likert scale methodology we employed in this study. In particular we note that the existence of a central 'indifferent' category may not be measuring the same trait [18]. However we elected not to force respondents into considering an item as either important or unimportant and conducted our analysis by discounting the indifferent category.

Response rates varied across the schools and were below the overall 50% which the project had aimed for. The challenge of getting good response rates from graduates is well recognised hence the decision by school A to utilise a face to face event to launch the survey which resulted in the best response rate. The student survey response rates were similarly low with no apparent difference between electronic or face to face strategies. It is therefore possible that data were skewed by the respondent population being particularly personally motivated by, or inherently more interested in the transition into the profession.

A further limitation of the study is the potential overlap and understanding of the terms used in the questionnaire. For example, problem solving and decision-making skills require a level of underpinning clinical knowledge. This aspect would be an area of interest for a future qualitative study.

## Discussion

A large number of attributes were considered either important or very important by both groups which is not surprising given that the attributes were derived from a combination of the day one competences and University of Edinburgh graduate attributes statements (Table 2). This consistency was also reflected in the qualitative data where similar themes emerged across both groups.

Focussing on attributes ranked as 'very important', highlights the importance to recent graduates of attributes viewed as non-technical. It has been argued recently that this terminology is unhelpful - not least because it defines what these attributes or skills are not rather than what they are and thus can, in some contexts become devalued [19]. Nevertheless the importance of attributes in this category is clear as is the understanding that graduate attributes, regardless of category, should be 'woven' into the curriculum not least because embedding in this way gives the potential for the attributes to extend beyond the original context in which they were acquired [4]. This notion is further supported by the strong evidence for the importance of integration of graduate attributes into the entire teaching and learning environment [20]. Furthermore such embedding is likely to give extra credibility to the attributes in the eyes of students and staff.

Further exploration of the qualitative data revealed a strong appreciation of the importance of communication skills in relationship building with clients. The study confirms that students and recent graduates appreciate the crucial importance of communication skills in terms both of client interactions and also with colleagues. Communication skills training is now well embedded in most veterinary curricula [21,22] (and in all 3 schools across which this study was carried out) where simulated clients are used in role play scenarios in small group formats. This study suggests that this is valued and if anything should be increased. The comparative analysis indicated that emphasising within this training the importance of compassion, friendliness and integrity in the practitioner/client interaction will be valuable to new graduates. The artificial nature of the role play scenarios used was mentioned in the qualitative data emphasising the importance of context and experiential learning which is described extensively in the medical education literature [23,24].

Practical skill acquisition was also a major concern to final year students and graduates with the students especially recognising the real variability that exists dependant on extramural studies experiences. The schools involved in this study all have clinical skills training facilities however it appears that this type of facility is not sufficient to address student concerns in this area. This is supported in studies by Walsh et al [25] and Heath and Mills [26] where employers also identified basic surgical skills as a potential area of weakness in graduates [26]. Whilst standardisation of clinical practical experiences is desirable, it is debatable whether the curriculum would ever be able to provide sufficient practical experience for students and recent graduates to feel comfortable and confident in this area. However the postgraduate development phase (PDP) has aimed in part to address this by guiding new graduates towards a set of 'Year One Competences' ideally with mentor support [3].

In comparison to other studies which focussed on the top rated attributes [27,28], our study allowed exploration of those attributes ranked less important. The analysis highlighted 3 attributes - business acumen, knowledge of veterinary practice management and research skills with significantly less agreement on the importance of these items on the questionnaire.

It was clear that articulating attributes relating to research and enquiry in the term 'research skills' led to interpretation of bench based laboratory work by some respondents which is likely to account in part for this result although other interviewees clearly understood the wider interpretation of the term. Nevertheless it appears that the word 'research' may have an 'image problem' amongst students and recent graduates. This is despite many initiatives and much political discussion globally to try and encourage more veterinary students to take an interest in research [29-31]. In a study specifically designed to consider research related graduate attributes, Laidlaw et al (2009) defined a set of 7 attributes common to research and clinical careers (Table 6) [25]. This methodology perhaps better explores the attributes underlying the term research skills.

**Table 6:** Comparison of top rated graduate attributes in this study with those identified in two related studies

**Non-academic attributes of good doctors**[27]	**Shared professional and research related attributes **[28]	Top 10 very important attributes - this study
**Delphi method**	**Interviews**	**Survey - recent graduates**

Recognition that patient care is the primary concern of a doctor	Inquiring mind/curiosity	Communication with clients and the public

Probity	Core knowledge	Communication with colleagues

Good communication and listening skills	Critical appraisal	Recognition of own limitations & knowing when to seek advice

Recognition of one's own limits and those of others	Understanding of the evidence base for professional practice	Listening skills

Pro-social attitude	Understanding of ethics and governance	Practical skills

Ability to cope with ambiguity, change, complexity and uncertainty	Ability to work in a team	Ability to cope with pressure

Commitment to lifelong learning	Ability to communicate	Problem solving

Compassion		Interpersonal and teamwork skills

Motivation and commitment		Decision making

Ability to be a team player		Compassion

The low ranking of business acumen and knowledge of veterinary practice management compared to the other attributes is interesting given the increased attention that these areas are currently being given in many veterinary curricula due in part to studies such as the Foresight report which considered the future of academic veterinary medicine [32]. It is interesting however that a recent study by Lane and Bogue indicated that veterinary faculty also ranked business skills relatively low [33]. It is evident from the qualitative data that graduates currently feel enough pressure in their new jobs focussing on the clinical aspects and perhaps that it is for other more experienced colleagues to focus on financial aspects. Nevertheless some business and financial awareness is desirable, not least as it exists as an explicit 'day one competency' [1] in new veterinary graduates but the content needs to be carefully considered in the context of the competing pressures on the curriculum and on the new graduate.

In addition to in-depth insight into the attributes considered important by final year students and recent graduates, this study also highlights some of the areas where opinions change or at least relative emphasis changes over this crucial transition period.

Knowledge of underpinning science was ranked relatively low in Table 2 by both cohorts. In addition, all other attributes where the word 'knowledge' appeared in the title (i.e. clinical knowledge, VPH, legislation, practice management were ranked significantly lower by the recent graduates (Table 4). In comparison to the study carried out by Laidlaw et al where core knowledge was ranked relatively high, in our study, veterinary clinical knowledge was ranked 15/42 by recent graduates and 7/42 by final year students when considered as a 'very important' attribute. This finding is interesting and consistent with opinion that a focus on knowledge is less important in current day universities [34]. The interviews identified a theme that knowing the common conditions then having the ability to seek further information elsewhere meant that extensive clinical knowledge was not as important as had been considered when a student. This appeared also to explain the difference in the two groups ranking of evaluating information.

It is interesting that the 'top 10' very important attributes as ranked by recent graduates included only one from the 'subject specific' category i.e. practical skills and was similar to the list identified in the study by Lambe et al [27] (Table 6). Comparing the attributes in this table also highlights similar attributes in the study by Laidlaw et al [28] although there are differences in the relative ranking of attributes.

## Conclusions

In conclusion, our study confirms the importance to final year veterinary students and recent veterinary graduates of non-technical attributes in the transition to working in the veterinary profession. Unsurprisingly perhaps, the appreciation of attributes which help foster the client/veterinarian relationship appear to become even more significant in recent graduates. At the same time, the graduates reflect that a focus on subject specific knowledge is less important once in practice when compared to final year. Recent graduates and final year students do not consider business acumen, knowledge of practice management and research skills as important as other attributes. It is anticipated that studies such as this will be able to inform curriculum developments and student support services in addition to professional development and support mechanisms for veterinarians in their post-graduate lives.

## Competing interests

The authors declare that they have no competing interests.

## Authors' contributions

SMR conceived the study, obtained the grant funding, drafted the manuscript, coordinated the data from University of Edinburgh graduates and students, conducted telephone interviews. SB and TK coordinated the data from Royal Veterinary College graduates and students, conducted telephone interviews. DJS combined datasets from the 3 institutions and performed the statistical analysis. CEB and RJM ran focus groups and gathered qualitative data. JH coordinated and obtained data from University of Glasgow. NPHH and REW coordinated data from University of Edinburgh graduates and students. RD informed design of survey with University of Edinburgh graduate attributes statements. All authors were members of the project steering group and read and approved the final manuscript.

## Pre-publication history

The pre-publication history for this paper can be accessed here:

http://www.biomedcentral.com/1472-6920/11/64/prepub

## Supplementary Material

Additional file 1 **Final Year student survey**. Survey completed by final year students either electronically or on paper. Click here for file

Additional file 2 **Recent Graduate survey**. Survey completed by recent graduates either electronically or on paper. Click here for file
