# Supplementary material for: The transition into veterinary practice: Opinions of recent graduates and final year students
Source: BMC Med Educ. 2011 Sep 22;11:64. doi: 10.1186/1472-6920-11-64 (PMC3188471; doi:10.1186/1472-6920-11-64)
Supplement: Additional file 1 — Final Year student survey. Survey completed by final year students either electronically or on paper. [file 1472-6920-11-64-S1.DOC]

**“Which skills and attributes help to ease the transition from veterinary student to new graduate/ clinician?”**

Veterinary curricula aim to provide new graduates with the necessary skills to be successful in the profession. Currently however little research exists which looks at the skills and attributes that recent graduates feel help to **ease the transition from student to new graduate**.

We would greatly appreciate 10 minutes of your time to complete this questionnaire so that we can explore this important issue further.

**Section A**

**General Information**

1. How old are you? _______ years

2. What sex are you?

- Male
- Female

3. Which vet school are you currently studying at?

School

- Edinburgh
- Glasgow
- RVC

4. Previous education

- Entered vet school from school
- Entered vet school after studying another University degree

**Section B**

**In your opinion, how important do you think the following skills and attributes are for**

**easing the transition between veterinary student and new graduate/ clinician?**

**Subject Specifi**c

|  | **Not at all**  **important** | **Not**  **important** | **Indifferent** | **Important** | **Very**  **important** |
| --- | --- | --- | --- | --- | --- |
| Veterinary  clinical knowledge | **☐** | **☐** | **☐** | **☐** | **☐** |
| Knowledge of  underpinning science | **☐** | **☐** | **☐** | **☐** | **☐** |
| Practical skills | **☐** | **☐** | **☐** | **☐** | **☐** |
| Knowledge of  veterinary practice  management | **☐** | **☐** | **☐** | **☐** | **☐** |
| Knowledge of  veterinary  legislation | **☐** | **☐** | **☐** | **☐** | **☐** |
| Knowledge of  veterinary public  health/  zoonotic issues | **☐** | **☐** | **☐** | **☐** | **☐** |

*Comments:*

General Skills And Attributes

|  | **Not at all**  **important** | **Not**  **important** | **Indifferent** | **Important** | **Very**  **important** |
| --- | --- | --- | --- | --- | --- |
| Business acumen | **☐** | **☐** | **☐** | **☐** | **☐** |
| Numeracy skills | **☐** | **☐** | **☐** | **☐** | **☐** |
| IT/ computer literacy | **☐** | **☐** | **☐** | **☐** | **☐** |

*Comments:*

Research & Enquiry

|  | **Not at all**  **important** | **Not**  **important** | **Indifferent** | **Important** | **Very**  **important** |
| --- | --- | --- | --- | --- | --- |
| Research skills | **☐** | **☐** | **☐** | **☐** | **☐** |
| Analytical skills | **☐** | **☐** | **☐** | **☐** | **☐** |
| Ability to evaluate  Information (e.g. in  marketing literature) | **☐** | **☐** | **☐** | **☐** | **☐** |

*Comments:*

Personal & Intellectual Autonomy

|  | **Not at all**  **important** | **Not**  **important** | **Indifferent** | **Important** | **Very**  **important** |
| --- | --- | --- | --- | --- | --- |
| Decision making | **☐** | **☐** | **☐** | **☐** | **☐** |
| Problem solving | **☐** | **☐** | **☐** | **☐** | **☐** |
| Thinking creatively and  independently | **☐** | **☐** | **☐** | **☐** | **☐** |
| Confidence | **☐** | **☐** | **☐** | **☐** | **☐** |
| Recognising own  limitations and knowing  when to seek advice | **☐** | **☐** | **☐** | **☐** | **☐** |
| Leadership skills | **☐** | **☐** | **☐** | **☐** | **☐** |
| Commitment to CPD | **☐** | **☐** | **☐** | **☐** | **☐** |

*Comments:*

**Communication skills**

|  | **Not at all**  **important** | **Not**  **important** | **Indifferent** | **Important** | **Very**  **important** |
| --- | --- | --- | --- | --- | --- |
| Communication with  clients and the public | **☐** | **☐** | **☐** | **☐** | **☐** |
| Communication with  colleagues | **☐** | **☐** | **☐** | **☐** | **☐** |
| Listening skills | **☐** | **☐** | **☐** | **☐** | **☐** |
| Negotiation skills | **☐** | **☐** | **☐** | **☐** | **☐** |
| Presentation skills | **☐** | **☐** | **☐** | **☐** | **☐** |
| Report writing and  record keeping skills | **☐** | **☐** | **☐** | **☐** | **☐** |

*Comments:*

Personal Effectiveness

|  | **Not at all**  **important** | **Not**  **important** | **Indifferent** | **Important** | **Very**  **important** |
| --- | --- | --- | --- | --- | --- |
| Flexibility in adapting to  new situations | **☐** | **☐** | **☐** | **☐** | **☐** |
| Interpersonal and  Teamwork skills | **☐** | **☐** | **☐** | **☐** | **☐** |
| Ability to cope with  uncertainty | **☐** | **☐** | **☐** | **☐** | **☐** |
| Ability to cope with  pressure | **☐** | **☐** | **☐** | **☐** | **☐** |
| Ability to handle  difficult situations | **☐** | **☐** | **☐** | **☐** | **☐** |
| Organisational skills | **☐** | **☐** | **☐** | **☐** | **☐** |
| Time management  skills | **☐** | **☐** | **☐** | **☐** | **☐** |
| Attention to detail | **☐** | **☐** | **☐** | **☐** | **☐** |
| Professional  appearance | **☐** | **☐** | **☐** | **☐** | **☐** |
| Compassion | **☐** | **☐** | **☐** | **☐** | **☐** |
| Patience | **☐** | **☐** | **☐** | **☐** | **☐** |
| Decisiveness | **☐** | **☐** | **☐** | **☐** | **☐** |
| Politeness | **☐** | **☐** | **☐** | **☐** | **☐** |
| Integrity | **☐** | **☐** | **☐** | **☐** | **☐** |
| Friendliness | **☐** | **☐** | **☐** | **☐** | **☐** |
| Ethical awareness | **☐** | **☐** | **☐** | **☐** | **☐** |
| Capacity for self-audit | **☐** | **☐** | **☐** | **☐** | **☐** |

*Comments:*

**In your opinion, which of the above are the THREE most important attributes in a veterinary surgeon?**


**Are there any additional attributes which you consider to be highly desirable in helping the transition from student to new graduate/ clinician?**


If you would be willing to participate in a focus group to discuss these issues further, please leave your contact details below

E-mail address:_____________________

Thank you for taking the time to complete this questionnaire - your input is much appreciated.
