# Supplementary material for: The transition into veterinary practice: Opinions of recent graduates and final year students
Source: BMC Med Educ. 2011 Sep 22;11:64. doi: 10.1186/1472-6920-11-64 (PMC3188471; doi:10.1186/1472-6920-11-64)
Supplement: Additional file 2 — Recent Graduate survey. Survey completed by recent graduates either electronically or on paper. [file 1472-6920-11-64-S2.DOC]

**“Which skills and attributes help to ease the transition from veterinary student to new graduate/ clinician?”**

Veterinary curricula aim to provide new graduates with the necessary skills to be successful in the profession. Currently however little research exists which looks at the skills and attributes that recent graduates feel help to **ease the transition from student to new graduate**.

We would greatly appreciate 10 minutes of your time to complete this questionnaire so that we can explore this important issue further.

**Section A**

**General Information**

1. How old are you? _______ years

2. What sex are you?

- Male
- Female

3. In which year and from which vet school and did you graduate? Year ___________

School

- Edinburgh
- Glasgow
- RVC

**4.** Before coming to vet school, what was your previous educational background?

- Entered vet school straight from school (A levels)
- Entered vet school straight from school (Scottish Highers)
- Entered vet school straight from school (International Baccalaureate)
- Entered vet school after studying another first degree in the UK
- Entered vet school after studying another first degree in North America
- Entered vet school after studying another first degree outside the UK or North America

5. Are you

- An assistant?
- A locum?
- A sole practitioner?
- Other? (Please give details)_____________________________

6. Do you currently do any clinical work?

- Yes, _______ % of my time (*Go to question 7*)
- No (*Go to Section B*)

7. What type of clinical work do you do? (*Please mark one response only*)

- Small animal only (may include some exotics)
- Farm animal only
- Equine only
- Laboratory animals only
- Exotic animals only (includes rabbits, small furries, reptiles, birds, zoo etc.)
- Mixed Practice (please indicate the percentage of time you spend on each discipline)

____% small animal ____% farm animal

____% equine ____% exotic

____% lab animal ____% meat hygiene

____% other (Please specify)___________________________

- Other (Please specify)______________________________________

**Section B**

**In your opinion, how important do you think the following skills and attributes are for**

**easing the transition between veterinary student and new graduate/ clinician?**

Subject Specific

|  | **Not at all**  **important** | **Not**  **important** | **Indifferent** | **Important** | **Very**  **important** |
| --- | --- | --- | --- | --- | --- |
| Veterinary  clinical knowledge | **☐** | **☐** | **☐** | **☐** | **☐** |
| Knowledge of  underpinning science | **☐** | **☐** | **☐** | **☐** | **☐** |
| Practical skills | **☐** | **☐** | **☐** | **☐** | **☐** |
| Knowledge of  veterinary practice  management | **☐** | **☐** | **☐** | **☐** | **☐** |
| Knowledge of  veterinary  legislation | **☐** | **☐** | **☐** | **☐** | **☐** |
| Knowledge of  veterinary public  health/  zoonotic issues | **☐** | **☐** | **☐** | **☐** | **☐** |

*Comments:*

General Skills And Attributes

|  | **Not at all**  **important** | **Not**  **important** | **Indifferent** | **Important** | **Very**  **important** |
| --- | --- | --- | --- | --- | --- |
| Business acumen | **☐** | **☐** | **☐** | **☐** | **☐** |
| Numeracy skills | **☐** | **☐** | **☐** | **☐** | **☐** |
| IT/ computer literacy | **☐** | **☐** | **☐** | **☐** | **☐** |

*Comments:*

Research & Enquiry

|  | **Not at all**  **important** | **Not**  **important** | **Indifferent** | **Important** | **Very**  **important** |
| --- | --- | --- | --- | --- | --- |
| Research skills | **☐** | **☐** | **☐** | **☐** | **☐** |
| Analytical skills | **☐** | **☐** | **☐** | **☐** | **☐** |
| Ability to evaluate  Information (e.g. in  marketing literature) | **☐** | **☐** | **☐** | **☐** | **☐** |

*Comments:*

Personal & Intellectual Autonomy

|  | **Not at all**  **important** | **Not**  **important** | **Indifferent** | **Important** | **Very**  **important** |
| --- | --- | --- | --- | --- | --- |
| Decision making | **☐** | **☐** | **☐** | **☐** | **☐** |
| Problem solving | **☐** | **☐** | **☐** | **☐** | **☐** |
| Thinking creatively and  independently | **☐** | **☐** | **☐** | **☐** | **☐** |
| Confidence | **☐** | **☐** | **☐** | **☐** | **☐** |
| Recognising own  limitations and knowing  when to seek advice | **☐** | **☐** | **☐** | **☐** | **☐** |
| Leadership skills | **☐** | **☐** | **☐** | **☐** | **☐** |
| Commitment to CPD | **☐** | **☐** | **☐** | **☐** | **☐** |

*Comments:*

**Communication skills**

|  | **Not at all**  **important** | **Not**  **important** | **Indifferent** | **Important** | **Very**  **important** |
| --- | --- | --- | --- | --- | --- |
| Communication with  clients and the public | **☐** | **☐** | **☐** | **☐** | **☐** |
| Communication with  colleagues | **☐** | **☐** | **☐** | **☐** | **☐** |
| Listening skills | **☐** | **☐** | **☐** | **☐** | **☐** |
| Negotiation skills | **☐** | **☐** | **☐** | **☐** | **☐** |
| Presentation skills | **☐** | **☐** | **☐** | **☐** | **☐** |
| Report writing and  record keeping skills | **☐** | **☐** | **☐** | **☐** | **☐** |

*Comments:*

Personal Effectiveness

|  | **Not at all**  **important** | **Not**  **important** | **Indifferent** | **Important** | **Very**  **important** |
| --- | --- | --- | --- | --- | --- |
| Flexibility in adapting to  new situations | **☐** | **☐** | **☐** | **☐** | **☐** |
| Interpersonal and  Teamwork skills | **☐** | **☐** | **☐** | **☐** | **☐** |
| Ability to cope with  uncertainty | **☐** | **☐** | **☐** | **☐** | **☐** |
| Ability to cope with  pressure | **☐** | **☐** | **☐** | **☐** | **☐** |
| Ability to handle  difficult situations | **☐** | **☐** | **☐** | **☐** | **☐** |
| Organisational skills | **☐** | **☐** | **☐** | **☐** | **☐** |
| Time management  skills | **☐** | **☐** | **☐** | **☐** | **☐** |
| Attention to detail | **☐** | **☐** | **☐** | **☐** | **☐** |
| Professional  appearance | **☐** | **☐** | **☐** | **☐** | **☐** |
| Compassion | **☐** | **☐** | **☐** | **☐** | **☐** |
| Patience | **☐** | **☐** | **☐** | **☐** | **☐** |
| Decisiveness | **☐** | **☐** | **☐** | **☐** | **☐** |
| Politeness | **☐** | **☐** | **☐** | **☐** | **☐** |
| Integrity | **☐** | **☐** | **☐** | **☐** | **☐** |
| Friendliness | **☐** | **☐** | **☐** | **☐** | **☐** |
| Ethical awareness | **☐** | **☐** | **☐** | **☐** | **☐** |
| Capacity for self-audit | **☐** | **☐** | **☐** | **☐** | **☐** |

*Comments:*

**In your opinion, which of the above are the THREE most important attributes in helping the transition from student to graduate/ clinician?**


**Are there any additional attributes which you consider to be highly desirable in helping the transition from student to new graduate/ clinician?**


If you would be willing to participate in a focus group to discuss these issues further, please leave your contact details below

E-mail address:_____________________

Thank you for taking the time to complete this questionnaire - your input is much appreciated.
